# The Transcription Factor Nrf2 Protects Angiogenic Capacity of Endothelial Colony-Forming Cells in High-Oxygen Radical Stress Conditions

**DOI:** 10.1155/2017/4680612

**Published:** 2017-05-15

**Authors:** Hendrik Gremmels, Olivier G. de Jong, Diënty H. Hazenbrink, Joost O. Fledderus, Marianne C. Verhaar

**Affiliations:** ^**1**^ Department of Nephrology and Hypertension, University Medical Center Utrecht, Heidelberglaan 100, 3584CX Utrecht, Netherlands; ^2^Department of Physiology, Anatomy and Genetics, University of Oxford, Oxford, UK

## Abstract

**Background:**

Endothelial colony forming cells (ECFCs) have shown a promise in tissue engineering of vascular constructs, where they act as endothelial progenitor cells. After implantation, ECFCs are likely to be subjected to elevated reactive oxygen species (ROS). The transcription factor Nrf2 regulates the expression of antioxidant enzymes in response to ROS.

**Methods:**

Stable knockdown of Nrf2 and Keap1 was achieved by transduction with lentiviral shRNAs; activation of Nrf2 was induced by incubation with sulforaphane (SFN). Expression of Nrf2 target genes was assessed by qPCR, oxidative stress was assessed using CM-DCFDA, and angiogenesis was quantified by scratch-wound and tubule-formation assays *Results.* Nrf2 knockdown led to a reduction of antioxidant gene expression and increased ROS. Angiogenesis was disturbed after Nrf2 knockdown even in the absence of ROS. Conversely, angiogenesis was preserved in high ROS conditions after knockdown of Keap1. Preincubation of ECFCs with SFN reduced intracellular ROS in the presence of H_2_O_2_ and preserved scratch-wound closure and tubule-formation.

**Results:**

Nrf2 knockdown led to a reduction of antioxidant gene expression and increased ROS. Angiogenesis was disturbed after Nrf2 knockdown even in the absence of ROS. Conversely, angiogenesis was preserved in high ROS conditions after knockdown of Keap1. Preincubation of ECFCs with SFN reduced intracellular ROS in the presence of H_2_O_2_ and preserved scratch-wound closure and tubule-formation.

**Conclusion:**

The results of this study indicate that Nrf2 plays an important role in the angiogenic capacity of ECFCs, particularly under conditions of increased oxidative stress. Pretreatment of ECFCs with SFN prior to implantation may be a protective strategy for tissue-engineered constructs or cell therapies.

## 1. Introduction

The formation of new vasculature is critical for tissue regeneration after ischemic injury. Postnatal neovascularization occurs classically through angiogenesis, the extension of preexisting vascular networks. Increasing evidence shows that de novo formation of vascular structures, vasculogenesis, also persists in the adult organism [1]. It is thought that a rare subset of progenitor cells, termed endothelial colony-forming cells (ECFCs), or late-outgrowth endothelial progenitor cells (late EPCs) resides within the vascular wall, which can be mobilized into circulation in response to vascular injury and contributes to the formation of new vessels [2]. ECFCs can be isolated from the blood of adults [3] as well as from cord blood [4] and can be expanded to large numbers for therapeutic applications [5]. The potential of ECFCs to organize into functional neovessels [6] has found practical applications both in cell therapy for ischemic vascular disease [7] and in tissue engineering [8, 9].

The tissue microenvironment in ischemic areas is characterized by an excess of reactive oxygen species (ROS), leading to oxidative stress [10]. This oxidative stress leads to the dysfunction of the endothelium and exacerbates vascular disease [11–13]. ECFCs are particularly sensitive to oxidative stress [14, 15], which may reduce their effectiveness as cell therapy or in tissue-engineered constructs.

The expression of endogenous cellular antioxidant defenses is regulated through a common promoter site called the antioxidant responsive element (ARE) [16, 17]. AREs, in turn, constitute a binding site for the transcription factor 2 (Nrf2) [18]. Nrf2 belongs to the cap'n'collar subset of basic leucine-zipper (bZip) transcription factors [18]. In the resting cell, Nrf2 is sequestered in the cytoplasm by its inhibitor, Kelch ECH-associating protein (Keap1), and is continuously targeted for ubiquitination [19]. Upon exposure to oxidative stress, Keap1 undergoes a conformational change and releases Nrf2, which can then translocate to the nucleus and initiate an ARE-dependent transcriptional response [18, 20]. Selective Nrf2/ARE activators can mimic this response by interacting with the same cysteine residues on Keap1 that are involved in ROS sensing [21].

The Nrf2/ARE regulatory network has an extensive number of conserved target genes involved in the oxidative stress response, cellular metabolism, and protein degradation, whose expression is partially tissue dependent [22]. The activation of Nrf2 reduces oxidative stress in endothelial cells [23] and suppresses inflammatory responses that may lead to cardiovascular disease. Due to the pleiotropic effects of Nrf2 activation, the causal contribution of different target genes is difficult to disentangle, but heme oxygenase 1 (HO-1) has been shown to play a major role in the antioxidant effects [24]. Nrf2 knockdown studies have shown that the loss of Nrf2 impairs tubule formation of endothelial cells on Matrigel [25, 26]. Age-induced impairments in Nrf2 activation are associated with atherosclerosis and vascular dysfunction [27–29], and treatments activating Nrf2 may attenuate the progression of atherosclerosis [29].

In the present study, we investigate the role of Nrf2 in ECFCs by examining the effects of Nrf2 pathway modulation on functional progenitor characteristics of ECFCs. Furthermore, we explore pretreatment options with the selective Nrf2 activator sulforaphane (SFN). We reason that Nrf2 activation via SFN primes the antioxidant defenses of ECFCs and consequently reduces their susceptibility to oxidative stress. This strategy may provide benefit in clinical therapeutic applications of ECFCs both in cell therapy and in tissue engineering.

## 2. Methods

### 2.1. Reagents

All reagents were purchased from Sigma-Aldrich (Zwijndrecht, NL) unless otherwise specified.

### 2.2. Cell Isolation and Culture

Human cord blood (50–90 ml) was collected from full-term pregnancies, in acid-citrate-dextrose anticoagulant blood collection bags as in [30]. The study protocol was approved by the local ethics committee (01/230 K, Medisch Ethische Toetsings Commissie (METC), University Medical Center Utrecht). Mononuclear cells (MNCs) were isolated by density-gradient centrifugation using Ficoll-Paque (GE Healthcare, Eindhoven, NL) and resuspended in EGM-2 growth medium (Lonza, Wakersville, MD, USA), containing SingleQuots (hEGF, VEGF, hFGF-2, Long R3-IGF-1, heparin, and gentamicin/amphotericin B), 10% fetal calf serum, and 100 units/100 *μ*g penicillin/streptomycin per ml. MNCs were then plated at a density of 2·10^6^ cells/cm^2^ on rat tail collagen I-coated wells (BD Biosciences, Bedford, MA) and cultured at 37°C, 5% CO_2_ in a humidified incubator. After 24 h, nonadherent cells were aspirated and complete EGM-2 medium was added to each well. Medium was replaced every other day thereafter. After 8–10 days, colonies with a typical cobble-stone morphology started to appear, and cells were harvested for further use upon reaching confluency. All experiments were conducted with cell passages 3–5, and multiple donor isolates were used for each experiments as indicated in the figure legends.

### 2.3. Lentiviral Constructs and Transduction

The ARE reporter construct was kindly provided by Dr. Hanna Leinonen of Kuopio University. It contains three sequential ARE sequences taken from the promoter region of the glutamate-cysteine ligase regulatory subunit (GCLM) gene, driving a firefly luciferase gene.

The plasmids containing short-hairpin ribonucleic acid (shRNA) against Nrf2 and Keap1 were purchased from Open Biosystems. 5 different shRNA sequences against the respective genes were used simultaneously in each experiment. The plasmids contain a puromycin resistance gene to facilitate selection.

Lentiviruses were produced using the ViraPower expression system. Plasmid DNA was complexed using polyethyleneimine (PEI) and added to a 15 cm dish containing 293 T cells. 48 hours after transfection, the virus containing supernatant was harvested, aliquoted, and stored at −80°C.

Lentiviral particles were charge neutralized using hexadimethrine bromide (Polybrene) and added to a T-25 flask containing ECFCs at 50% confluency. After 24 h, medium was replaced, and after 72 h, selection was initiated, during which cells were cultured in the presence of 2 *μ*g/ml puromycin for 7 days. This transduction procedure yields stable lines that show persistence of knockdown for multiple passages [31].

### 2.4. Chemical Nrf2 Activation

To assess Nrf2 activation as a pretreatment strategy, we used the Nrf2 activator l-sulforaphane (SFN) to induce the translocation of Nrf2 to the nucleus. ECFCs were incubated with different concentrations of l-sulforaphane (Sigma-Aldrich) for 4 hours unless otherwise specified, prior to exposure to H_2_O_2_.

### 2.5. Gene Expression by PCR

Total RNA was isolated using spin columns (RNAspin mini, GE Healthcare, Buckinghamshire, UK) and quantified by spectrophotometry (ND-1000, NanoDrop Technologies). First-strand cDNA was synthesized using the iScript™ cDNA Synthesis Kit (Bio-Rad, Hercules, CA) according to the manufacturer's instructions.

Specific primers for heme oxygenase (decycling) 1 (HO-1), NAD(P)H dehydrogenase [quinone] 1 (NQO1), glutamate-cysteine ligase catalytic subunit (GCLC), glutamate-cysteine ligase regulatory subunit (GCLM), and nuclear factor (erythroid-derived 2)-like 2/NF E2-related factor (Nrf2) were designed, as well as primers for several housekeeping genes (see Supplementary Table 1 available online at https://doi.org/10.1155/2017/4680612 for sequences). Primers were designed to work at an annealing temperature of 60 degrees Celsius.

The real-time PCR analysis was performed with iQ™ Sybr Green Supermix (Catalogue no. 170-8885, Bio-Rad, Hercules, CA), conducted according to the instructions of the manufacturer. Data were analyzed using the efficiency-corrected Delta-Delta Ct method [32]. The fold change values of the genes of interest (GOIs) were normalized using the geometric average of the fold change values of multiple housekeeping genes. The best housekeeping genes were selected by implementing the pairwise variance algorithm introduced in [33], using the geNorm applet (http://medgen.ugent.be/~jvdesomp/genorm/).

### 2.6. Western Blot

Cultured cells were lysed in modified RIPA buffer (50 mM Tris, 150 mM NaCl, 0.1% SDS, 0.5% sodium deoxycholate, 1% Triton X100, 1 mM EDTA, and complete protease inhibitors [Roche, Woerden, NL]), or nuclear extracts were made using the Nuclear Extract Kit by Active Motif. Total protein concentration was determined using the bicinchoninic acid (BCA) method (Pierce, Rockford, IL). Equal amounts of protein were loaded in a 4–12% SDS-PAGE gel (Life Technologies), the gel was set to run at 200 V, and protein was subsequently transferred to a polyvinylidene fluoride (PVDF) membrane.

After blocking, the membrane was stained with anti-Nrf2 (D1Z9, Cell Signaling, Leiden, NL) and developed using a chemiluminescent peroxidase substrate (Sigma-Aldrich). Ponceau staining was used to verify equal loading.

### 2.7. Oxidative Stress Measurements with CM-H_2_DCFDA

The effect of Nrf2 pathway modulation on intracellular ROS was determined using 5,6-chloromethyl-2′,7′-dichlorodihydrofluorescein diacetate, (CM-H_2_DCFDA, Life Technologies) [34]. ECFCs were incubated for 1 hour with 5 *μ*g/ml CM-H_2_DCFDA, washed once, and were allowed to recover for 30 minutes before being exposed to increasing concentration of H_2_O_2_. Intracellular fluorescent signal was measured on a fluorescent plate reader at Ex/Em 485/538 nm after 2 hours. Cell number was corrected for using PrestoBlue® cell viability reagent (Life Technologies, Bleiswijk, NL).

### 2.8. Scratch-wound Assays

To assess the horizontal ECFC migration and response to damage signals, a scratch-wound assay was performed [35]. ECFCs were grown until confluence in a 24-well plate. A scratch in the monolayer was made, detached cells were washed off, and a range of H_2_O_2_ dissolved in EGM-2 was added to the wells. Photographs were made on demarcated reference points at baseline and *t* = 6 hours. The average scratch width per high-powered field was calculated by dividing the area of the scratch by the length of the scratch, and migration was subsequently calculated by subtracting the width at baseline by the width at *t* = 6 hours.

### 2.9. Tubule Formation on Matrigel

Tubule formation on Matrigel was performed similarly to [30]. The inner well of an IBIDI *μ*-slide angiogenesis (IBIDI, Martinsried, Germany) was filled with 10 *μ*l growth factor-reduced Matrigel (BD). Next, 10^4^ ECFCs containing various constructs were suspended in 50 *μ*l EGM-2 containing different concentrations of H_2_O_2_ and laid on top of the Matrigel. Cells were allowed to form tubular networks for 6 hours, after which photographs were taken. A number of junctions were quantified using the freeware program AngioQuant [36].

Viability was examined by using the LIVE/DEAD® kit manufactured by Invitrogen.

### 2.10. Statistics

The results were analyzed by 1-way analysis of variance (ANOVA) in dose-response experiments. For pathway modulation and pretreatment experiments, analysis was performed by mixed linear models, allowing for random slopes within ECFC donor. Replicates indicate ECFCs from multiple human cord blood donors. All data are represented as means +/− standard error of the mean (SEM).

## 3. Results

### 3.1. The Effects of Nrf2 Pathway Modulation on Angiogenic Capacity of ECFCs

**Figure 1:**
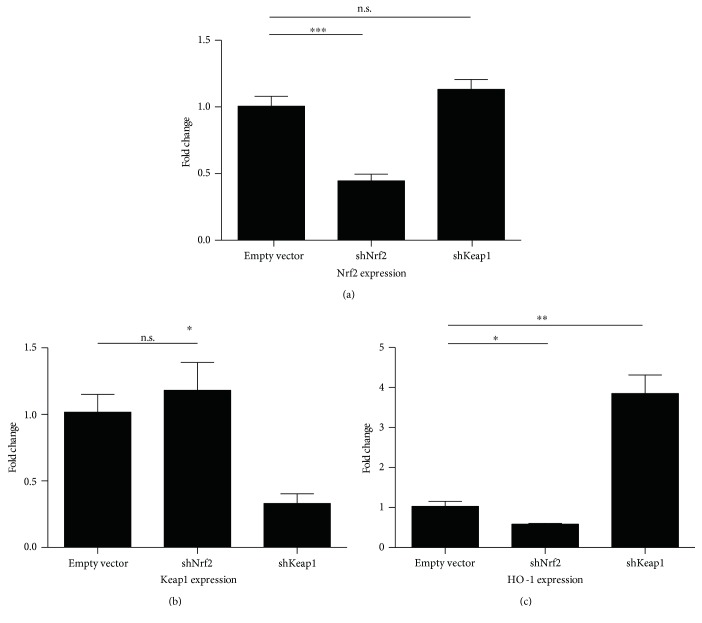
Nrf2 pathway modulation—effects on gene expression: (a) transduction with shRNAs against Nrf2 resulted in a significant reduction in Nrf2 expression (*p* < 0.001). No effect on Keap1 was observed. (b) shRNAs against Keap1 reduced its expression, (*p* = 0.02). (c) Effects of shRNAs on Nrf2/ARE target gene HO-1, shNrf2 transduction induced a reduction in HO-1 expression (*p* = 0.01) and shKeap1 increased HO-1 expression (*p* = 0.004), all experiments reflect data from 3 independent replicates.

To obtain more insight into the role of Nrf2 in ECFCs, we stably transduced ECFCs of different donors with lentiviral vectors expressing short-hairpin ribonucleic acids (shRNAs) against Nrf2 and against Keap1, the physiological inhibitor of Nrf2. Nrf2 knockdown efficiency was 60% using the lentiviral shRNA constructs (*p* < 0.001, Figure 1(a)). A similar efficiency of knockdown was observed with shKeap1 (*p* = 0.02, Figure 1(b)). Knockdowns were also reflected in expression of Nrf2/ARE target gene heme oxygenase 1 (HO-1), expression of which was reduced in shNrf2 (*p* = 0.02) and increased in Keap1 knockdown (*p* = 0.004, Figure 1(c)).

#### 3.1.1. Nrf2 Is Important in the Management of Intracellular ROS Levels

**Figure 2:**
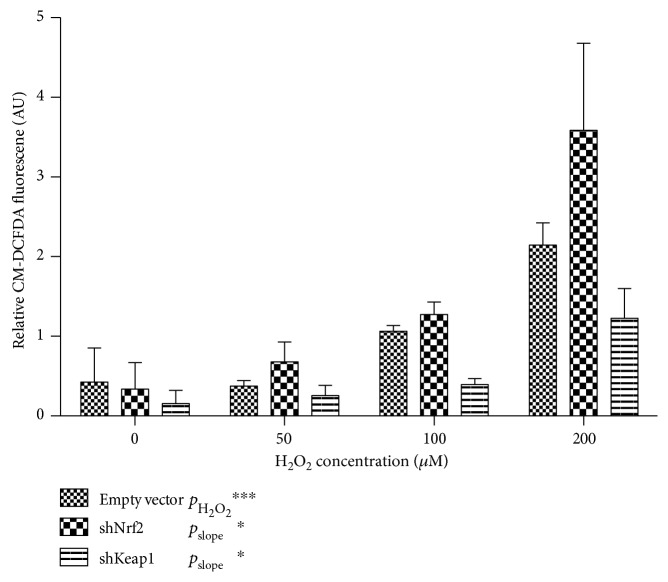
Nrf2 pathway modulation and oxidative stress: (a) intracellular ROS levels as measured by CM-H_2_DCFDA. ECFCs show a dose-dependent increase in CM-H_2_DCFDA signal in response to H_2_O_2_ (*p* < 0.001). Keap1 knockdown reduces oxidative stress in the presence of higher concentrations of H_2_O_2,_ (*p* = 0.04), whereas Nrf2 knockdown increases susceptibility to higher concentrations of H_2_O_2_ (*p* = 0.01). Graphs represent mean +/− SEM, and data are from 3 independent biological replicates.

To evaluate the effects of modulation of the Nrf2/ARE system on oxidative stress, we assessed intracellular ROS levels using the reporter dye CM-DCFDA to (Figure 2) Cells were loaded with CM-DCFDA for 30 min and exposed to increasing concentrations of H_2_O_2_. ECFCs showed a dose-dependent increase in intracellular ROS upon H_2_O_2_ exposure (*p* < 0.001). Knockdown of Nrf2 led to higher intracellular ROS levels at given concentrations of H_2_O_2_, (*p*_slope_ = 0.01) whereas knockdown of Keap1 led to very low-intracellular ROS levels, even in the presence of high concentrations of H_2_O_2_ (*p*_slope_ = 0.04).

**Figure 3:**
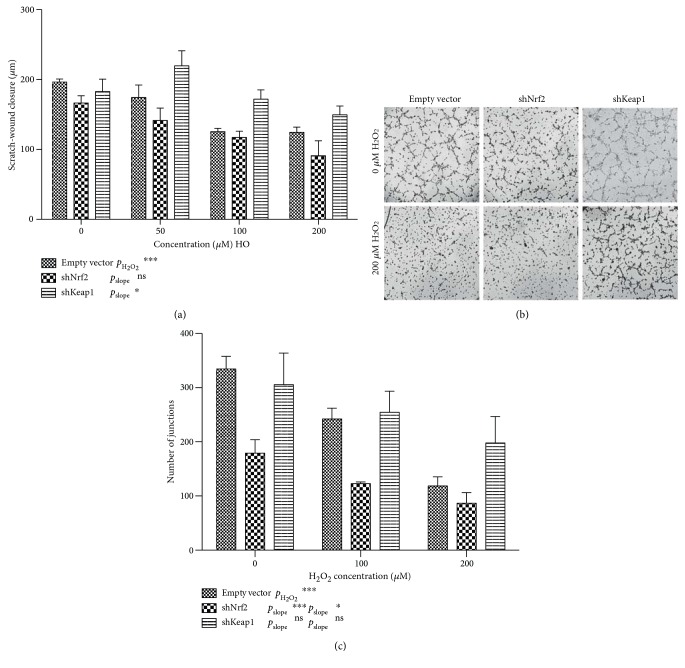
Nrf2 pathway modulation and endothelial function. (a) Endothelial scratch-wound closure in Nrf2 and Keap1 knockdown ECFCs in the presence of increasing concentrations of H_2_O_2_. Graphs represent mean +/− SEM, and data are from 4 independent biological replicates. (b) Tubule formation assay on Matrigel shows that tubule formation is sensitive to ROS. Nrf2 knockdown markedly impairs ECFC ability to form tubules even in the absence of H_2_O_2_, whereas Keap1 knockdown prevents H_2_O_2_-mediated inhibition of tubule formation. (c) Quantification of tubule formation by number of junctions. The number of junctions in the endothelial network greatly decreases in increasing concentrations of H_2_O_2_. Nrf2 knockdown significantly impairs tubule formation compared to control (*p* < 0.001), and Keap1 knockdown confers some resistance to H_2_O_2_-mediated impairment in tubule formation (*p* = 0.06). Graphs represent mean +/− SEM, and data are from 6 independent biological replicates.

*3.1.1.1. Nrf2 Is Involved in Angiogenesis*. We used a scratch-wound assay to assess how oxidative stress impairs ECFC function and the effects of Nrf2 thereupon. We observed a dose-dependent inhibition of scratch-wound closure under the influence of H_2_O_2_ (*p* < 0.001). No significant alterations were observed in scratch-wound closure upon Nrf2 knockdown, whereas knockdown of Keap1 resulted preservation of scratch-wound closure at elevated concentrations of H_2_O_2_ (*p* = 0.01, Figure 3(a)).

We next evaluated the effects of modulating the Nrf2/ARE system in the presence of oxidative stress on ECFC tubule formation on Matrigel. ECFCs transduced with different vectors were placed on Matrigel and exposed to increasing concentrations of H_2_O_2_. In the empty vector control, we observed a reduction in tubule formation in the presence of high concentrations of H_2_O_2_, with only minimal reticular organization of ECFCs at 200 *μ*M H_2_O_2_ (*p* < 0.001, Figures 3(b) and 3(c)). Knockdown of Nrf2 greatly reduced network formation even in the absence of H_2_O_2_, and no networks were formed in the presence of H_2_O_2_ (*p* < 0.001). Knockdown of Keap1 did not significantly preserve network formation in the presence of H_2_O_2_ (*p* = 0.06 for slope). Viability of shNrf2-transduced cells appeared to be preserved, even in high concentrations of H_2_O_2_ (Supplementary Figure 1).

### 3.2. Pretreatment Nrf2 Activator Sulforaphane to Reduce ECFC Susceptibility to Oxidative Stress

#### 3.2.1. Nrf2 Activation

**Figure 4:**
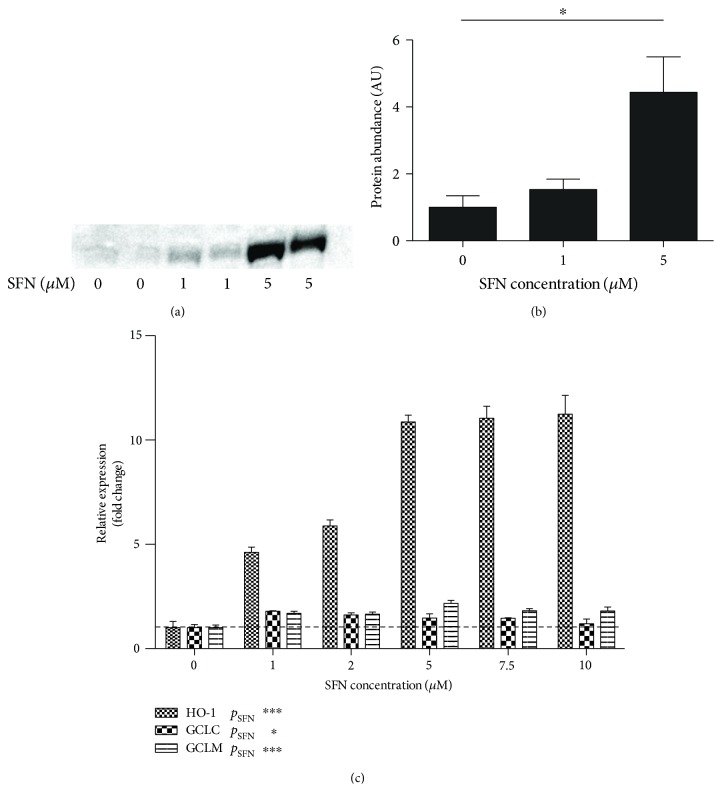
Nrf2 activation by SFN pretreatment in ECFCs. (a) Western blot of nuclear extracts of ECFCs exposed to SFN (Nrf2 band at ~72 kD). Data represent two donors. (b) Densitometric quantification of Western blot, showing dose-dependent increase of nuclear Nrf2 translocation (*p* = 0.02, *n* = 3) (c). Upregulation of Nrf2 target genes after incubation with sulforaphane (SFN). Glutamate-cysteine ligase expression, catalytic (GCLC) and regulatory subunit, is moderately upregulated in ECFCs treated with SFN. Heme oxygenase 1 (HO-1) shows a strong dose-dependent increase in expression with a plateau at ca. 5 *μ*M. Graphs represent mean +/− SEM, and data are from 3 independent biological replicates.

We evaluated the Nrf2/ARE system in a pretreatment strategy designed to guard ECFCs against oxidative stress. We used the selective Nrf2/ARE activator l-sulforaphane (SFN) to induce the nuclear translocation of Nrf2. To demonstrate the binding of Nrf2 to genomic ARE sequences, we used an ARE-driven luciferase reporter system, containing two ARE sequences from the promoter of the glutamate-cysteine ligase regulatory subunit (GCLM) gene. We stimulated the cells with the selective Nrf2/ARE activator l-sulforaphane (SFN). A significant time- and concentration-dependent increase in luciferase activity was observed after 4 hours, with a further increase to at least 24 h (Supplementary Figure 1). Further experiments were performed with 4-hour pretreatment. The nuclear translocation of Nrf2 showed a dose-dependent increase in response to SFN (*p* = 0.02) after 4 hours (Figures 4(a) and 4(b)), with a maximum of ca. 4× increased protein abundance. The nuclear translocation of Nrf2 was associated with increased expression of Nrf2 target genes heme oxygenase 1 (HO-1, *p* < 0.001), glutamate-cysteine ligase catalytic subunit (GCLC, *p* = 0.04) and glutamate-cysteine ligase regulatory subunit (GCLM, *p* < 0.001) (Figure 4(c)). Incidentally, the SFN-mediated upregulation of antioxidant gene expression is largely abrogated in Nrf2 knockdown (Supplementary Figure 2).

#### 3.2.2. SFN Preconditioning Preserves ECFC Function

**Figure 5:**
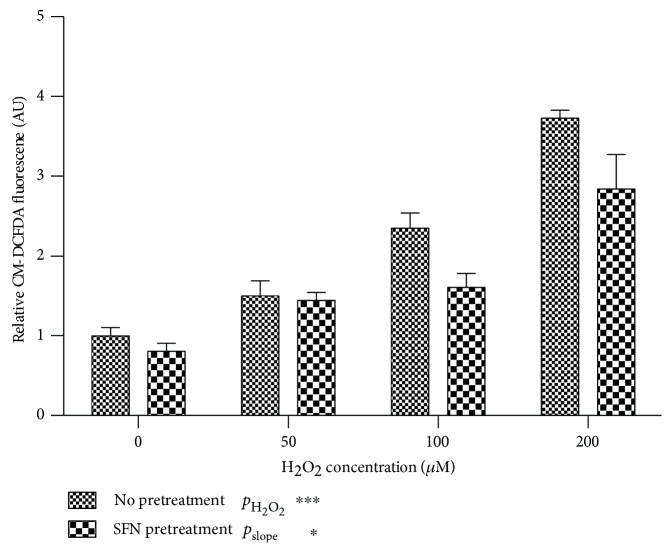
SFN pretreatment reduces ROS. Intracellular ROS as measured by CM-DCFDA fluorescence increased dose dependently with exposure to H_2_O_2_. Pretreatment of ECFCs with SFN ameliorated the H_2_O_2_-induced rise in ROS (*p* = 0.03). Graphs represent mean +/− SEM, and data are from 3 independent biological replicates.

To give a functional readout of antioxidant gene expression, we measured intracellular ROS with CM-H_2_DCFDA. A dose-dependent increase in CM-H_2_DCFDA fluorescence in the presence of increasing concentrations of H_2_O_2_ (*p* < 0.001) was observed. The preincubation of ECFCs with 2.5 *μ*M SFN reduced ROS levels, especially at high concentrations of H_2_O_2_ (*p* = 0.03, Figure 5).

**Figure 6:**
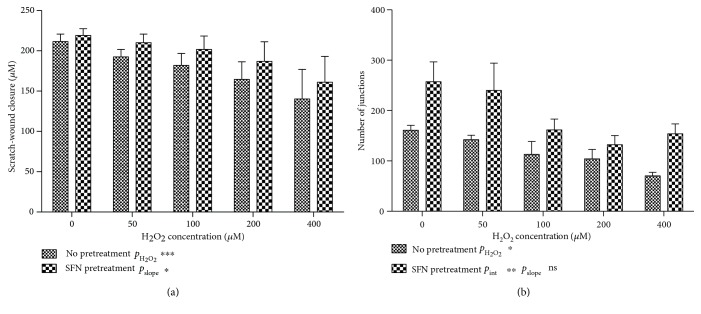
Pretreatment with SFN protects endothelial function. (a) Scratch-wound closure was reduced in the presence of increasing concentrations of H_2_O_2_. Pretreatment with 2.5 *μ*M SFN partially prevented the ROS reduction in migration (*n* = 6, *p* < 0.001). (b) SFN pretreatment increases numbers of junctions overall (*p* = 0.002), and no additional effect in the presence H_2_O_2_ is observed. Graphs represent mean +/− SEM, and 6 independent biological replicates were used for (a) and 4 replicates for (b).

Next, we assessed whether the preincubation of ECFCs with SFN could preserve angiogenic ability of ECFCs. We observed that the preincubation of ECFCs with 2.5 *μ*M SFN did not increase scratch-wound closure at baseline but partially preserved the reduction of migration in the presence of H_2_O_2_ (*p* < 0.001, Figure 6(a)). In the tubule formation assay, the preincubation with SFN showed an increased number of junctions overall (*p* = 0.002), but especially at low concentrations of H_2_O_2_. At higher concentrations of H_2_O_2_, tubule formation decreased at approximately the same rate as in non-pretreated cells (*p* = 0.69) but remained higher overall. Altogether, these data show a protective effect of SFN preincubation on ECFC function.

## 4. Discussion

In this study, we show that Nrf2 is important in the angiogenic response of ECFCs. The activation of Nrf2 by knockdown of Keap1 preserves cellular functions associated with angiogenesis, such as migration and tubule formation in the presence of ROS. The disruption of Nrf2 signaling inhibits these functions, even in the absence of ROS. Pretreatment with Nrf2/ARE activator SFN preserved ECFC function in conditions with high-oxygen radical stress. These findings suggest that Nrf2 is a potential target for modulation of ECFCs in their application as progenitor cell graft.

In the aging Western population, there is an increasing demand for regenerative medicine to extend the healthy life span. Progenitor cell therapy can be used to restore failing organs or tissues and thus delay functional decline. Endothelial progenitor cells have potential applications as separate therapy for vascular disease [37] and are an integral part in vascularized tissue-engineered organ constructs [8, 38]. ECFCs are a commonly investigated endothelial cell type, as they are easily obtained from peripheral blood even of adult patients [5] and have the capacity to form vascular networks [39]. Both in tissue engineering [30] and in cell therapy, [7] the survival of ECFC is important for the stability of the newly formed vessels. Studies investigating the cell fate of implanted cells using a luminescent p67^phox^ reporter system have shown that cells are exposed to a great amount of oxidative stress after implantation into ischemic tissue [10]. As ECFCs have been shown to be very susceptible to oxidative stress [14, 15], ex vivo pretreatment may be used to protect their in vivo function.

We confirm that moderate amounts of ROS can impair survival, migration, and angiogenic ability of ECFC, which are important for functioning in vivo [7]. Our data show that the activation of Nrf2 in human ECFCs by the knockdown of its inhibitor, Keap-1, limited the effects of oxygen radical stress and preserved endothelial function in an adverse environment. Previous studies [25] have shown an impairment in the migration and tubule-forming ability of coronary arterial endothelial cells upon siRNA knockdown of Nrf2. Kuang et al. [26] observed similar effects in the rat and showed increased susceptibility to hypoxia in Nrf2 knockdown cells. The protective effects of Keap1 knockdown can also be achieved by pretreating the cells with the Nrf2/ARE activator SFN. SFN is an isothiocyanate compound isolated from broccoli that is a strong inducer of ARE-driven genes [40]. It has been administered to patients in several clinical trials and has an excellent safety profile [41–43].

In this study, upon knockdown of Nrf2, tubule formation was profoundly reduced, even in the absence of ROS. These findings have been observed previously [24–26] and are consistent with a role for redox signaling in angiogenesis. In a similar experiment, Florczyk et al. have shown that this phenotype could be partially reversed by overexpression of HO-1, underlining the importance of this enzyme in the endothelial response to ROS [24]. ROS have previously been implicated in the signal transduction of angiogenic growth factors such as VEGF and angiopoietin-1 [44–46]. VEGF stimulation has been shown to increase ROS production though NAD(P)H oxidase activation in endothelial cells, leading to phosphorylation of VEGFR2 [47]. Impairments in growth factor signaling, in particular in IGF-2 and HIF1a, have been observed in Nrf2 knockout animals. [48, 49]. This suggests a common role for Nrf2-induced antioxidant enzymes in orchestrating redox signaling events, by controlling the activity of the serine/threonine kinase domain of growth factor receptors [25, 48]. In addition to limiting the effects of ROS on vascular signaling and angiogenesis, Nrf2 has also been shown to have a direct role in augmenting vascular sprouting by regulating tip cell formation via interaction with the Notch/Dll4 system [50]. These results suggest that there may be an advantage to transient Nrf2/ARE activation to allow for sprout formation but that a subsequent reduction in Nrf2 activation is required for vessel maturation.

The present study has several limitations: the ECFCs used were obtained from cord blood and not from adult peripheral blood as they would for clinical applications. There are indications that functional differences in angiogenic ability exist between neonatal and adult ECFCs that are related to increased senescence [39]. Vascular ageing has been shown to be associated with deficient activation of the Nrf2/ARE system in response to oxidative stress [29], which may limit pretreatment strategies. A further limitation is that in the present study, the effects of ex vivo Nrf2 modulation in ECFCs are only shown in isolated in vitro model systems. Angiogenesis is complicated multifaceted process, involving a complex interplay of endothelial cells, pericytes, and myeloid cells. For instance, in tissue-engineered constructs containing ECFCs, influx of host myeloid cells is required for vessel formation [51]. The importance of investigating the role of Nrf2 also in vivo is illustrated by the paradoxical fact that Nrf2 −/− animals show increased angiogenesis after ischemic injury, in contrast to the reduction in angiogenesis observed in vitro after Nrf2 knockdown [24]. This is likely secondary to an increased inflammatory response in Nrf2 −/− animals, with concomitant rise in influx of myeloid cells. To separate the effects of Nrf2/ARE activation on endothelium versus the systemic angiogenic response, pretreatment strategies should be applied ex vivo on the cell graft only, rather than a systemic preconditioning of the patient. Further research is however required to substantiate the findings presented here and to investigate potential interactions of Nrf2 modulation in ECFCs with the transplant recipient.

A further advantage of activating Nrf2 ex vivo, rather than administering an Nrf2/ARE activator systemically, is that chronic Nrf2/ARE activation has been associated with increased tumor progression and vascularization [52]. However, in patients with pre-existing malignant disease, extreme caution should be applied in administering progenitor cell constructs in the first place [53].

In summary, the present study confirms the role of Nrf2 in angiogenesis and shows its protective role against oxidative stress in ECFCs. These results indicate that Nrf2 modulation, for example with SFN, may be a pretreatment strategy to enhance the function of ECFCs in cell therapy and tissue-engineering applications.

## Supplementary Material

Table 1. Primer sequences. Figure 1. Viability stain of ECFCs at 6 hours after initiation of a tubule forming experiment on matrigel. Green cells are alive, whereas red cells are dead. While Nrf2 knockdown disrupts tubule formation, viability is preserved, even in high concentrations of H_2_O_2_. Figure 2. Time- and Dose-dependent ARE activation: ECFCs of three donors were transduced with a luciferase-reporter constuct driven by three GCLM promoter sequences (lower panel). Luciferase signal after incubation with different doses of Nrf2 pathway activator Sulforaphane (SFN) was measured at different time-points over the course of 24h. Figure 3. Effects of Nrf2 and Keap1 knockdown on gene expression. Nrf2 and Keap1 expression is not modulated by SFN. Significant knockdown was achieved by shRNA transduction (p<0.05 in both shNrf2 and shKeap1). HO-1 expression is increased after exposure to SFN (p<0.001), which is greatly reduced with Nrf2 knockdown. Knockdown of Keap1 increased HO-1 expression at baseline to an elevated level and no further upregulation of gene expression was observed in response to SFN stimulation. Graphs represent mean +/− S.E.M., data represent 3 independent biological replicates.
