# Supplementary material for: The Transcription Factor Nrf2 Protects Angiogenic Capacity of Endothelial Colony-Forming Cells in High-Oxygen Radical Stress Conditions
Source: Stem Cells Int. 2017 May 15;2017:4680612. doi: 10.1155/2017/4680612 (PMC5451769; doi:10.1155/2017/4680612)
Supplement: Supplementary file 1 — Table 1. Primer sequences. Figure 1. Viability stain of ECFCs at 6 hours after initiation of a tubule forming experiment on matrigel. Green cells are alive, whereas red cells are dead. While Nrf2 knockdown disrupts tubule formation, viability is preserved, even in high concentrations of H2O2. Figure 2. Time- and Dose-dependent ARE activation: ECFCs of three donors were transduced with a luciferase-reporter constuct driven by three GCLM promoter sequences (lower panel). Luciferase signal after incubation with different doses of Nrf2 pathway activator Sulforaphane (SFN) was measured at different time-points over the course of 24h. Figure 3. Effects of Nrf2 and Keap1 knockdown on gene expression. Nrf2 and Keap1 expression is not modulated by SFN. Significant knockdown was achieved by shRNA transduction (p<0.05 in both shNrf2 and shKeap1). HO-1 expression is increased after exposure to SFN (p<0.001), which is greatly reduced with Nrf2 knockdown. Knockdown of Keap1 increased HO-1 expression at baseline to an elevated level and no further upregulation of gene expression was observed in response to SFN stimulation. Graphs represent mean +/− S.E.M., data represent 3 independent biological replicates. [file 4680612.f1.pdf]

Supplementary Information

Gene	Direction	Sequence	Tm (ºC)
GCLC	Forward	TCCTGGACTGATCCCAATTC	60
GCLC	Reverse	TGCGATAAACTCCCTCATCC	60
GCLM	Forward	GTTGGGATACTGTGGGCTCT	60
GCLM	Reverse	CGGCCCTGAATTAAGGATTT	60
HO-1	Forward	ACATCTATGTGGCCCTGGAG	60
HO-1	Reverse	CTGGTGTGTAGGGGATGACC	60
Keap1	Forward	GTGTGGAAAGAGCAGGCTTC	60
Keap1	Reverse	TGGGAACCACATTTCCAGAG	60
NQO1	Forward	TACTATGGGATGGGGTCCAG	60
NQO1	Reverse	TCTCCCATTTTTCAGGCAAC	60
Nrf2	Forward	GAGAGCCCAGTCTTCATTGC	60
Nrf2	Reverse	TTGGCTTCTGGACTTGGAAC	60
VEGF	Forward	GCCTTGCTGCTCTACCTCCA	60
VEGF	Reverse	CAAGGCCCACAGGGATTTT	60
CXCR4	Forward	TTTTCTTCACGGAAACAGGG	60
CXCR4	Reverse	GTTACCATGGAGGGGATCAG	60
Catalase	Forward	TTTCCCAGGAAGATCCTGAC	60
Catalase	Reverse	ACCTTGGTGAGATCGAATGG	60
eNOS	Forward	CTCATGGGCACGGTGATG	60
eNOS	Reverse	ACCACGTCATACTCATCCATACAC	60
Cathepsin L	Forward	TGGCCTAATGGATTATGCTTTCC	60
Cathepsin L	Reverse	ATGACCTGCATCAATAGCAACA	60
EMMPRIN	Forward	TTCACTACCGTAGAAGACCTTGG	60
EMMPRIN	Reverse	GTTGATGTGTTCTGACGACTTCA	60
IL-8	Forward	GAATGGGTTTGCTAGAATGTGATA	60
IL-8	Reverse	CAGACTAGGGTTGCCAGATTTAAC	60
**Housekeeping Genes**			
PPIA	Forward	ATGGTCAACCCCACCGTGT	60
PPIA	Reverse	TCTGCTGTCTTTGGGACCTTGTC	60
P0	Forward	TGCACAATGGCAGCATCTAC	60
P0	Reverse	ATCCGTCTCCACAGACAAGG	60
HPRT1	Forward	TGACACTGGCAAAACAATGCA	60
HPRT1	Reverse	GGTCCTTTTCACCAGCAAGCT	60

Table 1: **Primer sequences**

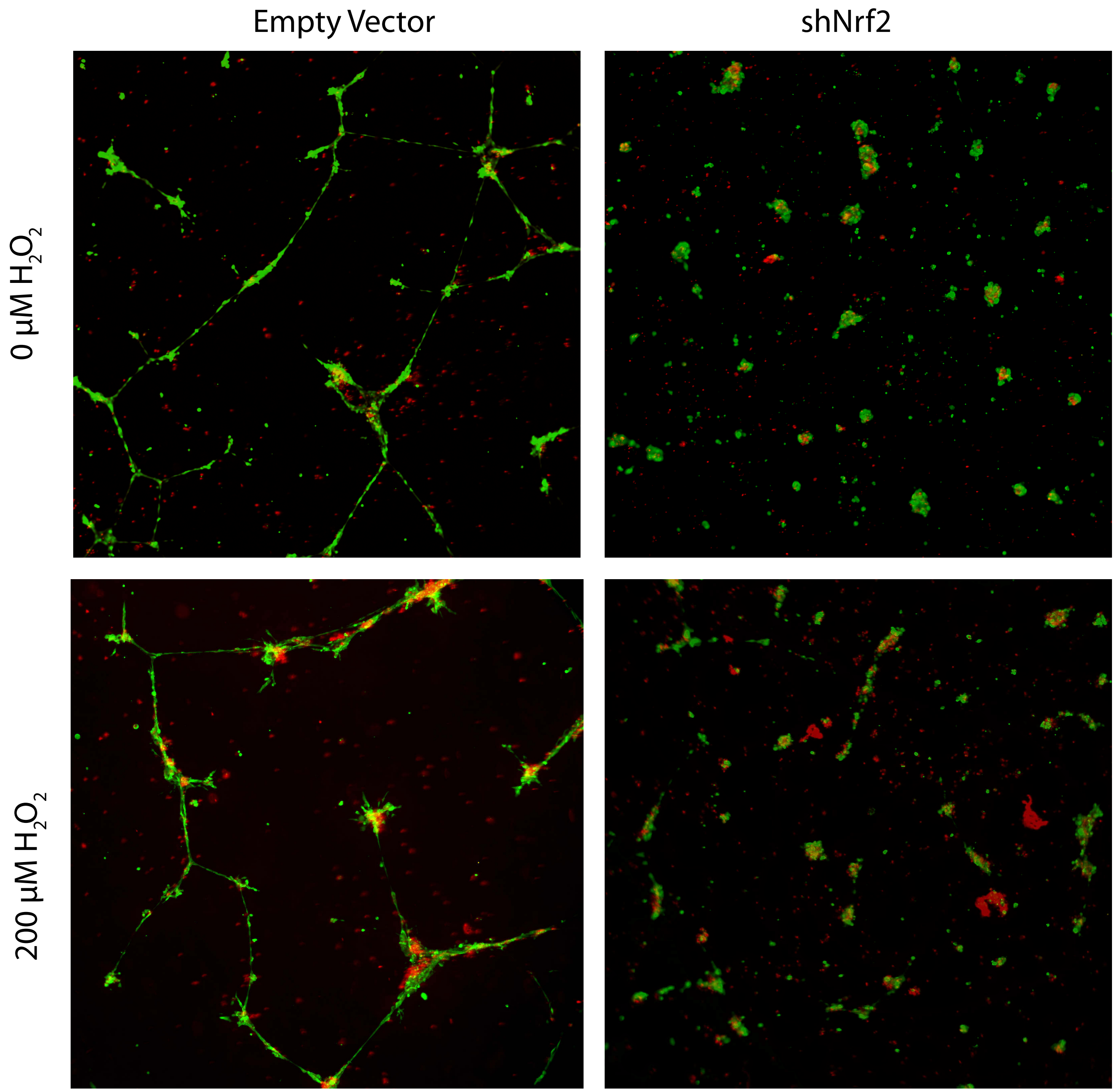

Figure 1: Viability stain of ECFCs at 6 hours after initiation of a tubule forming experiment on matrigel. Green cells are alive, whereas red cells are dead. While Nrf2 knockdown disrupts tubule formation, viability is preserved, even in high concentrations of H_2O_2

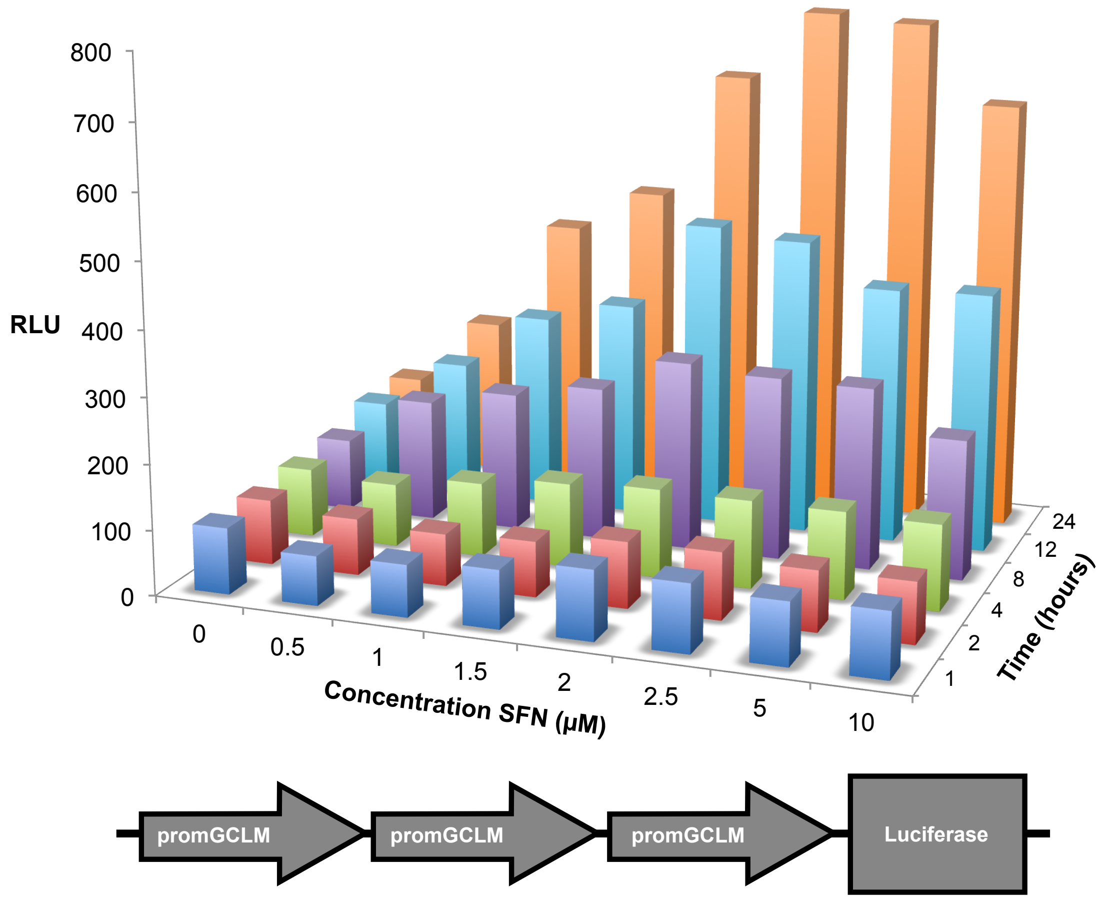

Figure 2: **Time- and Dose-dependent ARE activation:** ECFCs of three donors were transduced with a luciferase-reporter constuct driven by three GCLM promoter sequences (lower panel). Luciferase signal after incubation with different doses of Nrf2 pathway activator Sulforaphane (SFN) was measured at different time-points over the course of 24h.

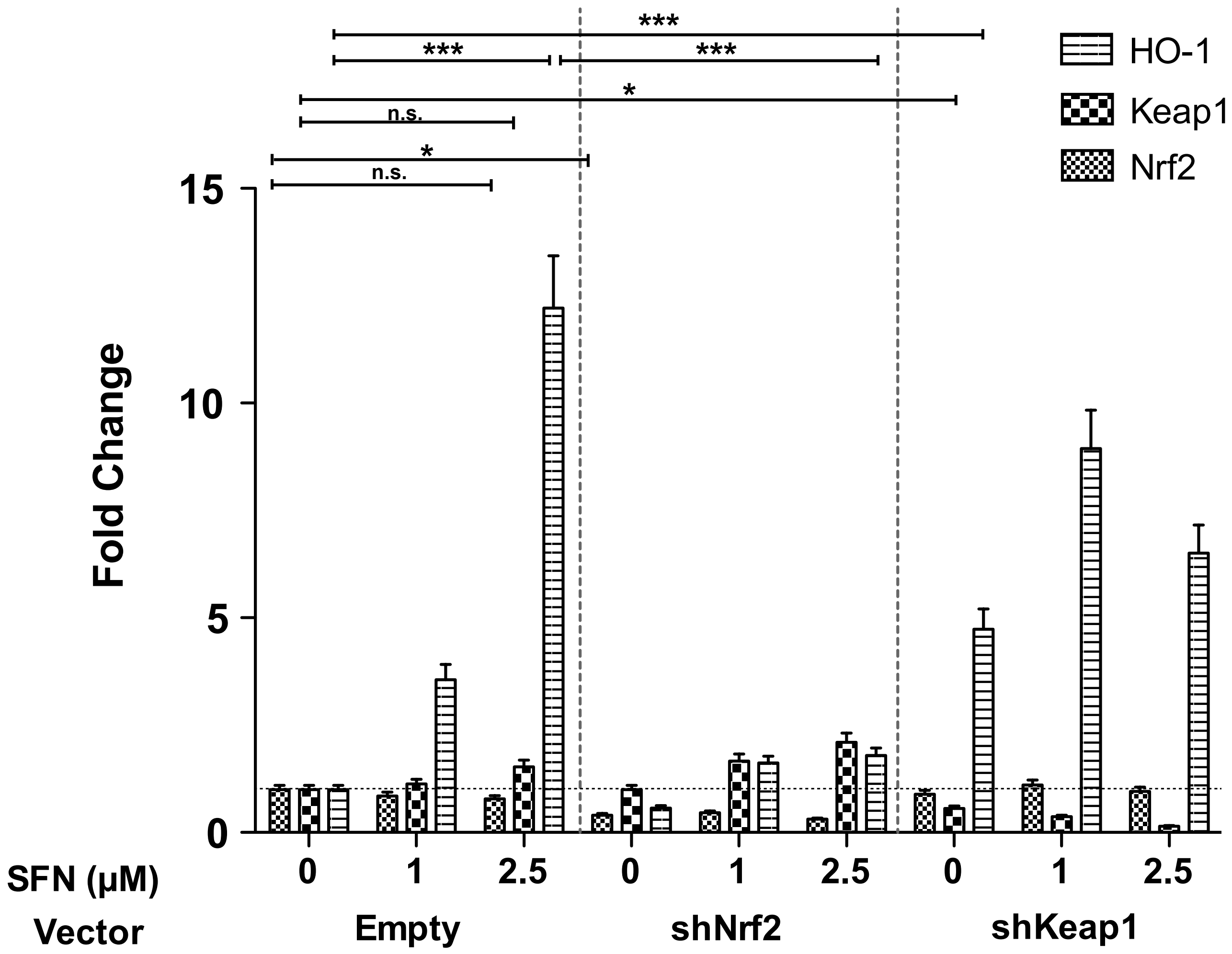

Figure 3: **Effects of Nrf2 and Keap1 knockdown on gene expression.** Nrf2 and Keap1 expression is not modulated by SFN. Significant knockdown was achieved by shRNA transduction ($p<0.05$ in both shNrf2 and shKeap1). HO-1 expression is increased after exposure to SFN ($p<0.001$), which is greatly reduced with Nrf2 knockdown. Knockdown of Keap1 increased HO-1 expression at baseline to an elevated level and no further upregulation of gene expression was observed in response to SFN stimulation. Graphs represent mean +/- S.E.M., data represent 3 independent biological replicates.
